# Elizabeth M. Miller, Thicker than water: A social and evolutionary study of iron deficiency in women

**DOI:** 10.1093/emph/eoaf005

**Published:** 2025-02-26

**Authors:** Masako Fujita

**Affiliations:** Department of Anthropology, Michigan State University, East Lansing, MI 48824, USA

*Thicker Than Water* is a masterful narrative of iron deficiency in women through a rare integration of ideas from evolutionary medicine, biological anthropology, and medical/sociocultural anthropology. It presents an enlightened analysis of why women are vulnerable to iron deficiency and makes a convincing case that the combination of evolved biology and global inequalities is the major culprit.

Thousands of maternal deaths are attributed to iron deficiency and/or anemia every year. The widespread belief is that this vulnerability is biologically inevitable because menstrual blood loss drains women of iron. Miller makes a case that iron loss in menstrual bleeding accounts for a meager proportion of iron loss and does not cause iron deficiency anemia under normal circumstances. Rather, the evolved set point for iron homeostasis is lower in reproductive-aged women than in men as a safety measure against harmful effects of excessive iron on her reproductive outcomes, posits Miller. In her view, this lower iron set point is adaptive and not pathologic as long as women have access to adequate dietary iron. The book traces the root causes of low dietary iron to food beliefs and practices that disadvantage women’s consumption. This is exacerbated by the fact that reproductive-aged women’s iron stores can be depleted through repeated pregnancies, which require high iron investment. In societies where women have low reproductive autonomy, their vulnerability to iron deficiency is heightened with increasing parity as iron expenditure exceeds iron replenishment through the diet.

The book reviews diverse bodies of literature, ranging from anthropology to evolutionary biology, to evolutionary medicine, to history of science/medicine. Miller uses life history theory to frame iron as a scarce resource, to be allocated between current and future reproduction. This adds to the work of Katie Hinde on rhesus milk calcium and phosphorus [1] and my work on human milk vitamin A [2] to demonstrate the value of a life history approach to female micronutrient metabolism beyond simple questions of energy allocation. Miller weaves her journey as a researcher, starting in her dissertation fieldwork years collecting data from women of northern Kenya, her years as junior faculty publishing these data, and trying to make sense of journal reviewers’ critiques and comments, through more recent work with NHANES data among US women. The book is not just a review of the literature on iron deficiency. It is a theory-informed feminist critique of the existing literature. It provides a new hypothesis that advances our understanding of how iron deficiency can be so common in women and yet be dismissed as biological inevitability.

The book starts with evolutionary perspectives and on the fundamentals of iron nutrition—what iron is, what it does for the human body, how it is needed for reproduction, how too little or too much can harm, and how a person’s iron nutrition can be characterized. Iron nutrition is complex and understanding it starts with teasing apart the chemistry of iron that makes it vital for life, yet harmful in excess. The book explains that iron absorption and blood levels of iron are tightly regulated to both harness iron’s chemical properties and limit their harm. Miller rightly expresses dismay that this fact is underappreciated in the current biomedical and public health approaches to iron nutrition in women.

The book then proposes that the human body has an evolved capacity for homeostatic control of iron. It further argues that, under normal circumstances and in reproductive-aged women, a fairly low iron set point avoids the harmful effects of excess iron, which can be devastating for women’s reproductive success, including fetal loss. Subsequent chapters build this understanding of women’s low iron as an adaptive default for female reproduction and explain why women’s iron often becomes too low to maintain their health, finding culprits in a modern dietary environment and food production and distribution. It also clarifies how iron absorption involves the health of our gut microbiome. Drawing on the concept of embodiment from critical medical anthropology, it shows how social inequalities “gets under the skin” via the gut. Another excellent idea from Miller is that embodiment allows us to conceptualize of how modern environments “become part of the human phenotype.” This makes the evolutionary medicine concept of “modern environment” more sophisticated and helps us link evolved bodies, a social world, and ill health. The latter chapters expand the scope to broader social contexts like sexism and racism that increase women’s vulnerability to iron deficiency and affect large swaths of women across the globe.

Chapter 4 entitled “Out for Blood” is one of my favorites. It provides a critique of biomedicine that has long viewed menstruation as an ill-designed culprit for women’s low iron. “We should be stating that menstruation is a sign of health, and that normal menstrual iron loss leaves no lasting pathology.” Miller walks us through three evolutionary hypotheses for women’s lower iron levels: a mismatch between evolved biologies and modern environment; a side effect (spandrel) hypothesis; and an adaptive hypothesis. The adaptive hypothesis is clearly the best supported and Miller shows how generations of scholars have it wrong. In patriarchal societies, the unspoken assumption is that male is default. Women’s unique physiology, like menstruation or lower iron, must be pathological: “our first instinct was to blame menstrual periods for women’s low iron.” Miller then skillfully overlays the sociocultural and political economy lens and describes how iron circulates from inorganic in the environment to organic in food (and humans) and then back to inorganic, how limited amounts of iron flow to, or from, certain populations of women due to the inequality in the distribution of nutritious food, clean water, hygiene, and sanitation.

This book would be an excellent read for evolutionary biologists and public health researchers interested in broad biosocial models of human health. It provides an exemplary contextualization of “modern environments” and mismatch hypotheses in an understanding of sociocultural processes such as sexism and racism.

I would use this book in my Human Adaptability course as a side reader. It would make a useful resource for students pursuing biological anthropology or critical medical anthropology or anywhere in between. Iron deficiency is the leading micronutrient deficiency that claims mothers’ lives in low income and wealthy nations alike, particularly women of color. This big problem, as the author eloquently argues, requires big ideas. Anthropology’s big ideas, drawn from different subfields, give powerful, holistic explanations that encompass both biological and cultural processes. My favorite sentence is, “Biological anthropologists can crack open the black box of the body as it is conceived in social theory” such as embodiment. Miller’s integration of evolutionary ecological lenses with the notion of embodiment of iron deficiency forms an excellent teaching tool to train a new generation of researchers who will ask the right questions and contribute to a holistic understanding of human biocultural processes that produce health inequalities toward more enlightened solutions.

## References

[1] Hinde K , Foster AB, Landis LM et al Daughter dearest: sex-biased calcium in mother’s milk among rhesus macaques. Am J Phys Anthropol 2013;151:144–50. DOI: https://doi.org/10.1002/ajhb.21195 23446791

[2] Fujita M , Shell-Duncan B, Ndemwa P et al Vitamin A dynamics in breastmilk and liver stores: a life history perspective. Am J Hum Biol 2011;23:664–73. DOI: https://doi.org/10.1002/ajhb.21195 21695742 PMC3938187

